# EpCAM (CD326) is differentially expressed in craniopharyngioma subtypes and Rathke’s cleft cysts

**DOI:** 10.1038/srep29731

**Published:** 2016-07-19

**Authors:** Vivian Thimsen, Annett Hölsken, Michael Buchfelder, Jörg Flitsch, Rudolf Fahlbusch, Harald Stefanits, Marco Losa, David T. W. Jones, Rolf Buslei

**Affiliations:** 1 Department of Neuropathology, Friedrich-Alexander University Erlangen-Nürnberg (FAU), Erlangen, Germany; 2 Department of Neurosurgery, Friedrich-Alexander University Erlangen-Nürnberg (FAU), Erlangen, Germany; 3 Department of Neurosurgery, University Clinic Hamburg-Eppendorf, Hamburg, Germany; 4 Department of Neurosurgery, International Neuroscience Institute, Hannover, Germany; 5 Department of Neurosurgery, Medical University of Vienna, Vienna, Austria; 6 Department of Neurosurgery, Ospedale San Raffaele, Milano, Italy; 7 Division of Pediatric Neurooncology, German Cancer Research Center (DKFZ), Heidelberg, Germany

## Abstract

The epithelial cell adhesion molecule (EpCAM) is a type I glycoprotein located on the surface of epithelial cells. It is strongly expressed in many neoplasms and already used in the diagnosis and distinction of various tumour subtypes. Comparative studies about EpCAM expression in cystic sellar lesions are lacking. Therefore, we analysed its distribution pattern in adamantinomatous (aCP) and papillary (pCP) craniopharyngiomas (CP) and Rathke’s Cleft Cysts (RCC) using immunohistochemistry and gene expression profiling. Whereas the protein was not detectable in pCP (n = 10), all aCP (n = 64) showed distinct staining patterns. The vast majority of RCC (n = 10) also appeared positive, but these displayed notably lower labeling scores. Additionally, significantly higher mRNA levels were detectable in aCP (n = 19) when compared to pCP (n = 10) (*p* = *9.985*^−*8*^). Furthermore, pediatric aCP cases, in general, exhibited stronger EpCAM staining levels compared to adult ones (*p* = *0.015*). However, we were not able to verify this result on mRNA level. In summary, our findings demonstrate that EpCAM can be used as an additional distinction-marker for cystic lesions of the sellar region. Its unknown function in aCP and the presence of an approved monoclonal bispecific trifunctional antibody for cancer therapy are interesting starting points for further studies.

Arising along the site of the vestigial craniopharyngeal duct, craniopharyngioma (CP) represent 2–5% of all primary intracranial neoplasms and are located in the sellar region^1^,^2^. CP are one of the most common non-neuroepithelial CNS lesions in children (0–14 years of age), accounting for 1.5–11.6% of tumours reported in this age group but represent only a minority of intracranial tumours in adults^3^. Despite the fact that these are declared as histologically benign grade I tumours according to WHO guidelines^4^, CP have devastating and life-threatening consequences due to their location near important brain structures like the hypothalamus, the pituitary gland and the optic chiasm^2^,^5^. Malignant transformations are extremely rare^6^,^7^. CP are a morphologically and clinically heterogeneous group of tumours that must be divided in two distinct subtypes: the more common adamantinomatous CP (aCP), which evince an increased tendency to relapse and occur mainly during childhood, and the rare papillary CP (pCP) variant, being almost exclusively found in adults and showing a more favorable clinical course.

aCP are characterised by a manifold histological appearance comprised of broad strands of multistratified squamous epithelium with peripheral palisading of nuclei, keratin nodules (“wet keratin”), cells with translucent nuclei (“ghost cells”), and distinct regressive tissue changes (e.g. organising hemorrhages, lymphocytic infiltrations, calcifications and cholesterol crystals). They also feature finger-like tumour outgrowths, making neurosurgical dissociation from surrounding brain tissue extremely challenging. In contrast, pCP appear more homogeneous, solid, and are usually not calcified^8^.

A correct classification is important because therapeutic treatment and prognosis are different for CP subtypes and other cystic non-neoplastic sellar lesions like Rathke’s Cleft Cysts (RCC)^9^,^10^,^11^,^12^. To date, several molecular differentiation markers like claudin-1^13^, EGFR-P^14^, Map2^15^ and nuclear β-catenin accumulations^16^,^17^ have been established. Additional genetic aberrations such as activating *CTNNB1* mutations in aCP^18^,^19^ and *BRAFV600E* mutations in pCP^20^,^21^,^22^ have also been observed, further supporting the importance of different signaling pathways in the pathogenesis of CP subtypes. In particular, activated Wnt signaling^23^,^24^,^25^,^26^, as well as SHH and EGFR pathways have been suggested to play pivotal roles in aCP^8^,^27^, whereas the activation of the MAPK pathway seems to be essential for pCP^28^. These findings not only serve for pathological differentiation, but they also represent potential targets for future therapy^29^,^30^.

The discovery of those molecular markers has supported appreciable progress in the correct diagnosis of sellar tumours. Nevertheless, limitations still exist, these being caused by the restricted specificity of some antibodies, particularly on small tissue specimens with limited tumour content, or by the comparably high costs of molecular analyses. For these reasons, additional immunohistochemical markers would be helpful. EpCAM (CD326) expression has already been observed in aCP^31^. It is a single-pass type I glycoprotein of 33–40 kDa with a long extracellular (EpEX) domain, a single transmembrane region, and a 26 amino acid short intracellular tail (EpICD)^32^. The EpEX contains an epidermal growth-like factor (EGF) and a predicted thyroglobulin (TY) domain. In the 1970’s, EpCAM was the first human tumour-associated antigen identified with monoclonal antibodies^33^. Ever since, strong EpCAM expression has been observed in various epithelial-derived tumours (e.g. breast cancer, colorectal cancer or lung cancer), precursor cells, and embryonic stem cells^34^,^35^,^36^,^37^. In fact, normal human epithelia express EpCAM, albeit to a significantly lower extent^32^. EpCAM is named after its most well described function as a homophilic Ca^2+^ -independent cell-cell adhesion molecule^38^,^39^, but is now recognised to be involved in other processes including cell signaling, migration, differentiation, proliferation, and tumour metastasis^40^,^41^. At the same time, EpCAM not only mediates cell-cell contacts but also weakens E-cadherin adhesions via the PI3 kinase pathway^39^,^42^,^43^. It further acts as a signaling molecule by regulated intramembrane cleavage and nuclear translocation, initiating the transcription of Wnt target genes like c-myc or cyclin-D1^44^,^45^,^46^, and influences renewal of epithelial cells by inhibition of TGF-β^41^. As it is also known to be a helpful marker in the distinction of various tumour entities^42^,^47^, we aimed to precisely describe EpCAM expression in a representative number of aCP, pCP and RCC specimens.

## Results

### Differential Immunohistochemical Distribution Pattern of EpCAM in Sellar Lesions

**Figure 1:**
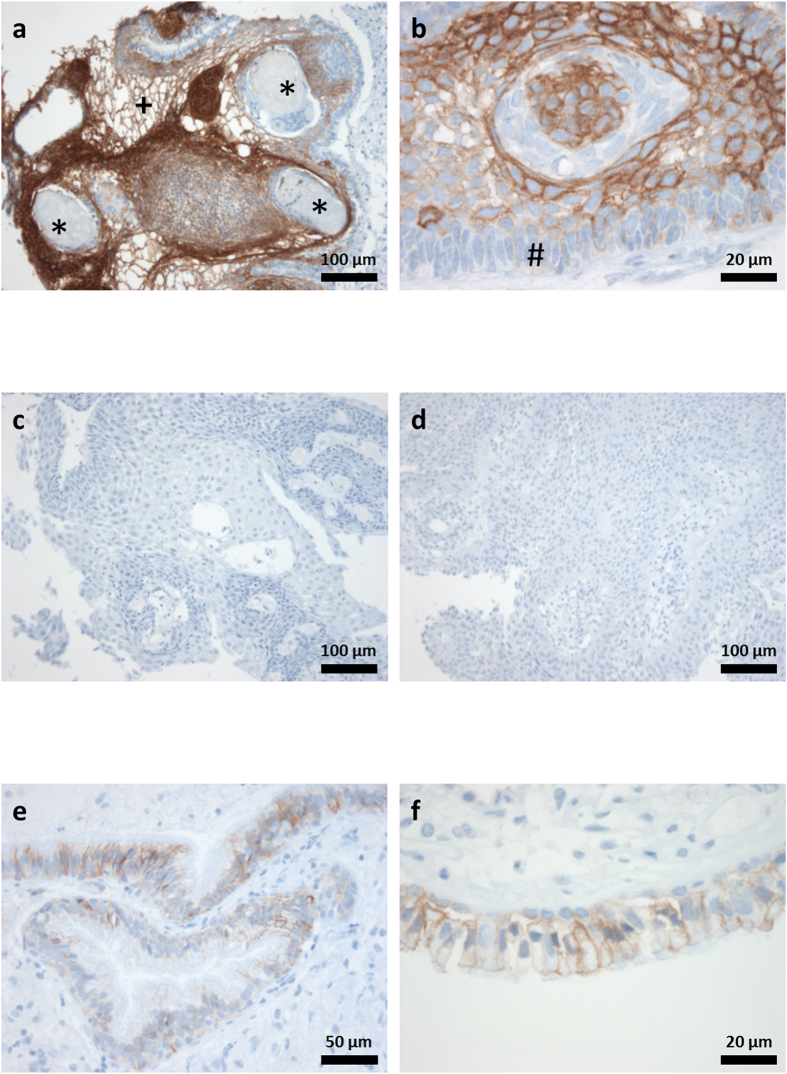
EpCAM Distribution Pattern in Lesions of the Sellar Region. (**a**,**b**) aCP showed a strong staining pattern especially in areas of stellate reticulum (+) and surrounding regressive changes (e.g. wet keratin (*)) and pale or no staining in cells of the basal cell layer (#). (**a**) aCP47, which was scored TIS = 9 (group S3). (**b**) aCP4, which was scored TIS = 9 (group S3). (**c**,**d**) Examples of pCP (pCP7 and pCP9) without any detectable staining, both scored into group S1. (**e**,**f**) RCC (RCC1) displayed a weak to moderate staining in the columnar epithelium which was scored TIS = 2 (group S2).

**Figure 2:**
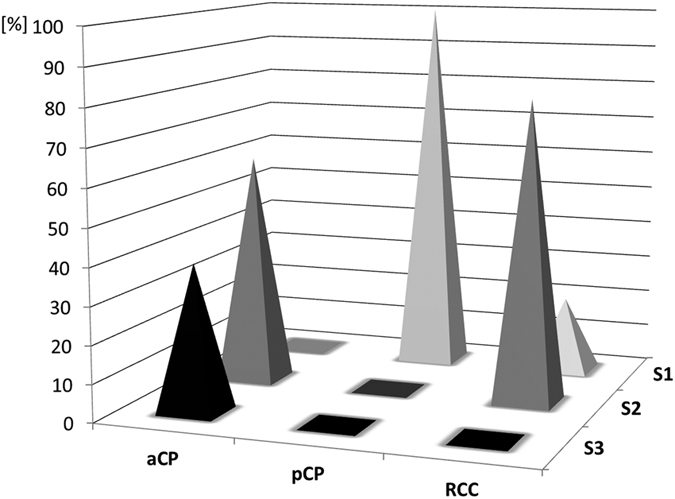
Summary of Calculated EpCAM Staining Scores Within the Different Groups of Lesions. The spikes illustrate the percentage shares of the immunohistochemical scoring groups (S1 = “no expression”; S2 = “low/moderate expression”; S3 = “strong expression”) within the groups of aCP, pCP, and RCC specimens under study. Whereas all aCP samples showed EpCAM expression in varying intensities (61% in group S2 and 39% in group S3), no immunostaining was detectable in the group of pCP (100% in group S1). In RCC, 20% of the samples showed no staining corresponding to group S1, while 80% revealed weak staining (group S2). Exact values for each tumour sample are given in Supplementary Table 1.

The distribution pattern of EpCAM was estimated using immunohistochemistry. For each case, a semi-quantitative total immunostaining score (TIS) was defined as described in detail in the methods section of this manuscript. Based on the calculated TIS, all specimens were subsequently divided into three different scoring groups (S1: TIS < 0; S2: TIS = 1–4; S3: TIS > 4). Representative examples from each group are given in Fig. 1. Although EpCAM staining was detectable in all aCP tumour samples (Fig. 1a,b), no specific immunoreaction was visible in the group of pCP (Fig. 1c,d). In RCC the vast majority revealed a certain antibody reaction (80%; Fig.1e,f). Overall, 39 aCP cases (61%) showed low or moderate EpCAM expression levels (S2) whereas 25 tumours (39%) exhibited a strong antibody reaction (S3). The respective scoring groups for each tumour entity are presented in Fig. 2.

**Figure 3:**
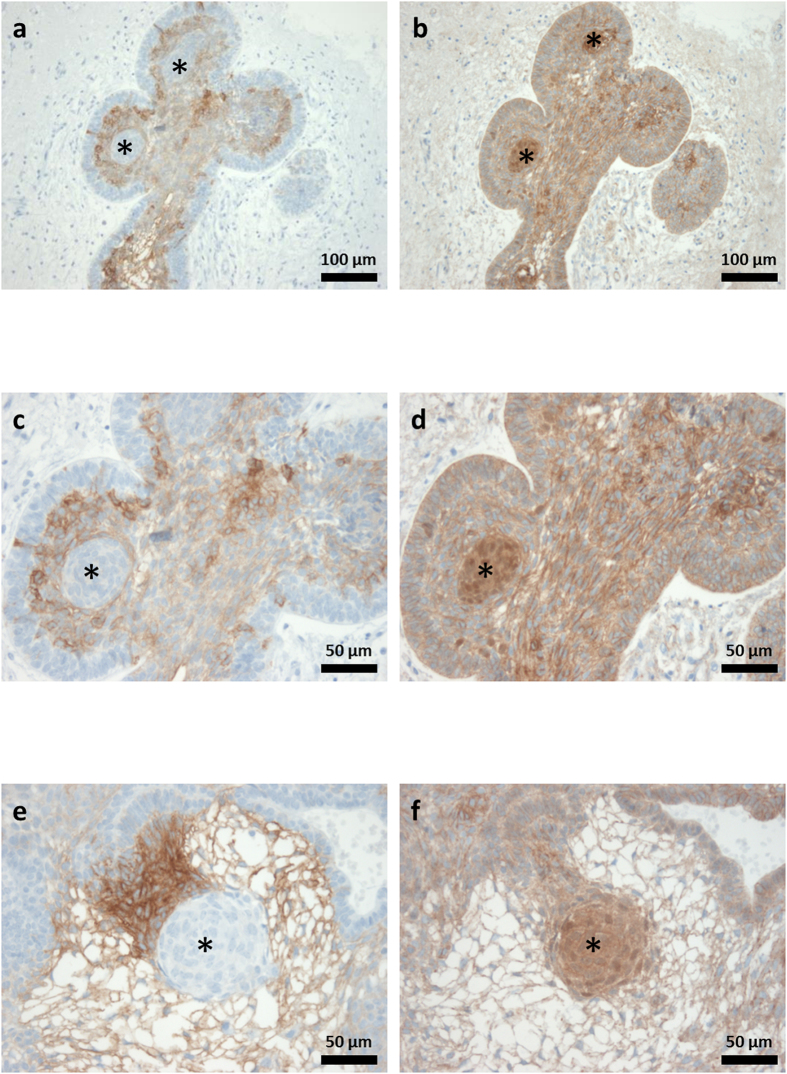
Immunohistochemical Staining of EpCAM and β-catenin in Paraffin Embedded Serial Sections of aCP. Immunohistochemical staining of serial sections using antibodies against EpCAM (**a**,**c**,**e**) and β-catenin (**b**,**d**,**f**) showed the absence of EpCAM in cell clusters with nuclear β-catenin accumulations (*).

Upon reviewing the positive cases, it became apparent that the staining intensity was not equally distributed throughout the tumour tissue. In aCP specimens, EpCAM immunoreactivity could always be detected at the cell membrane, in areas of stellate reticulum surrounding regressive changes (e.g. wet keratin, ghost cells, cholesterol deposits) (Fig. 1a,b). Interestingly, whirl-like cell clusters with tumour cells showing nuclear β-catenin accumulations completely lacked EpCAM expression. In contrast, adjoining cells were characterised by a strong and homogenous expression as documented in serial sections (Fig. 3a–f). Tumour cells of the basal layer, bordering on adjacent brain tissue, revealed only weak staining or remained completely negative.

In RCC, immunoreactivity was restricted to the cell membrane of the ciliated cuboidal cell-layer, whereas the cilia exhibited no EpCAM expression. Overall, when compared to the group of aCP, both staining intensities and quantity of labeled cells were noticeably lower. For this reason, all positive RCC samples were categorised in S2 representing low to moderate expression levels (Fig. 1e,f) accordingly.

Samples containing certain amounts of surrounding tissue revealed weak but homogeneous EpCAM immunostaining within the anterior pituitary gland. This predominantly appeared at the cell membrane of endocrine cells (Supplementary Material: S-Fig. 1a,b), confirming the observations of Ortiz-Plata *et al*.^48^. In contrast, neuronal and glial cells always remained completely negative (data not shown).

The respective staining results for BRAF(VE1), EpCAM and β-catenin for each sample included in this study can be found in Supplemental Material (S-Table 1).

### The Amount of EpCAM Staining in aCP was Associated with Age

**Figure 4:**
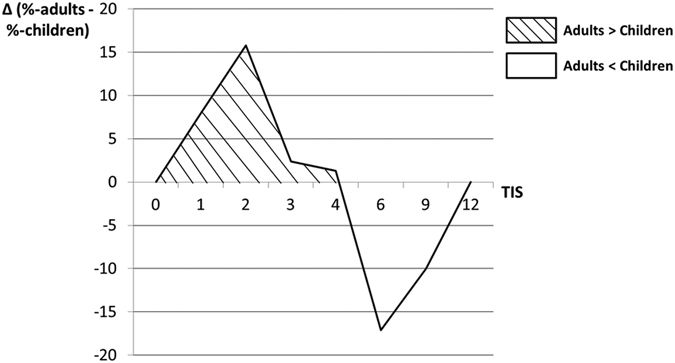
EpCAM Staining Scores in Relation to Patient Age. Tumours in adult patients showed predominantly lower EpCAM protein expression, and were, therefore, more strongly represented in TIS 1–4 (S2). In contrast, tumours in pediatric patients mainly showed a higher EpCAM protein expression, reflected in a higher representation of TIS > 4 (S3) in this age group. The number of adult and pediatric patients of each TIS was set in relation to the total number of patients of each age group. A comparative presentation was achieved by subtraction: 
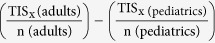
. As there was no tumour sample with TIS = 8, it was neglected in this graph. Possible TIS-values are 0, 1, 2, 3, 4, 6, 8, 9, and 12, resulting from the multiplication of intensity (0–3) and proportion (0–4) of EpCAM staining.

To address a potential prognostic value of EpCAM expression for patient care, the calculated staining scores were matched with several clinicopathological characteristics. Interestingly, a shift towards a younger age group was noticeable in the strong expression group (S3; Table 1). We set up a cross table (age groups: children 0–18 ys. vs. adults >18 ys. and scoring groups: S2 = low/moderate levels vs. S3 = strong levels) to examine statistical significance. This revealed a significant difference of EpCAM protein expression between the groups, with significantly higher staining in children [n(adults) = 41; n(children) = 23; Fisher’s exact test (two-sided): *p* = *0.015*]. To attain a comparative representation, the delta value was calculated by subtracting the individual percentages of children for each TIS from those of the individual adult cases. Thereby TIS = 1–4 (S2) is stronger represented in adults, while TIS > 4 (S3) had a higher incidence in children (Fig. 4).

We also investigated potential relationships between EpCAM expression and gender [n(female) = 25; n(male) = 39; Fisher’s exact test (two-sided): *p* = *0.298*], as well as invasiveness [n(invasive) = 19; n(non-invasive) = 22; Fisher’s exact test (two-sided): *p* = *0.330*], but the level of significance (p < 0.05) was not reached.

As the available clinical data concerning recurrences among our patient cohort was not extensive enough, we were not able to draw a reliable conclusion.

### EpCAM is Expressed Significantly Higher in aCP Compared to pCP

To verify the protein data, we compared EpCAM gene expression levels in both CP subtypes (19 aCP and 10 pCP) utilizing Affymetrix U133 Plus2.0 expression array and performed differential gene expression analysis. RNA was attained from snap frozen tissue samples with sufficient tumour content for a reliable profile.

**Figure 5:**
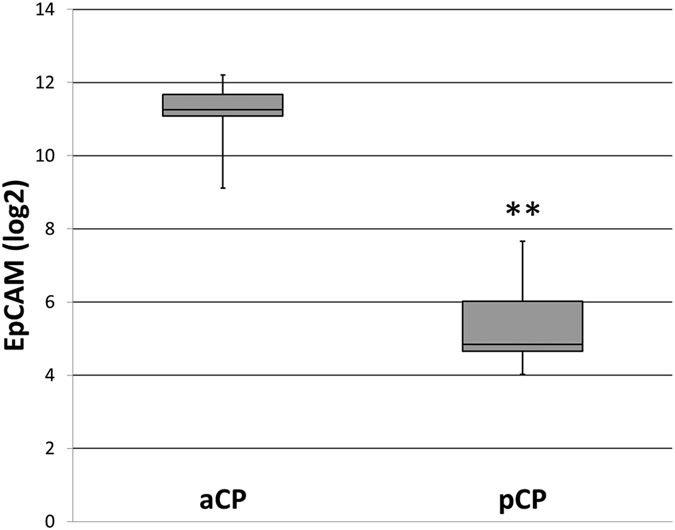
Relative EpCAM Expression (log2-ratio) in CP Subtypes. The Affymetrix U133 Plus2.0 expression array data (in log2 expression units) revealed significant differences in EPCAM gene expression between aCP and pCP. Therefore, aCP specimens (n = 19; median log2-ratio = 11.265; standard deviation (σ) = 0.684; range (R) = 3.09) exhibited significantly higher EpCAM levels compared with the group of pCP (n = 10; median log2-ratio = 5.336; σ = 1.159; R = 3.65) (Mann-Whitney test: *α* ≤ *0. 01; p* = *9.985*^−*8*^). Error bars are used to indicate the distribution range of single values.

Because Gaussian distribution could not be assumed after conducting the Kolmogorov-Smirnov and Shapiro-Wilks test, we compared the gene expression levels (log2-ratio) in both groups using the Mann-Whitney test for independent samples. Therefore, aCP specimens (n = 19; median log2-ratio = 11.265; standard deviation (σ) = 0.684; range (R) = 3.09) exhibited significantly higher EpCAM levels compared with the group of pCP (n = 10; median log2-ratio = 5.336; σ = 1.159; R = 3.65) (Mann-Whitney test: *α* ≤ *0. 01; p* = *9.985*^−*8*^; Fig. 5).

To assess the data concerning age-dependent EpCAM expression on the mRNA level, we additionally compared the average log2-ratio of aCP in children (n = 8; medium log2-ratio = 11.231; σ = 0.550; R = 1.690) and adults (n = 11; medium log2-ratio = 11.289; σ = 0.793; R = 3.056), but were not able to show a significant difference (Mann-Whitney test: *α* ≤ *0. 05; p* = *0.310*).

## Discussion

Monoclonal antibodies against epithelial cell adhesion molecules (EpCAM, CD326) were introduced in the late 1970’s^33^, representing the first molecular markers for colorectal cancer. Since then, EpCAM turned out to be a well-established and widely used diagnostic tool for human carcinomas^34^,^35^,^36^,^37^. Today it is frequently utilised as a differentiation marker for non-epithelial and epithelial derived neoplasms and it has also been identified as a potential targed for cancer therapies since appropriate anti-EpCAM-antibodies (e.g. Adecatumumab and Catumaxomab) are available^32^,^42^. EpCAM was previously described to be expressed in pituitary neoplasms^49^ were it can be helpful in distinguishing between different adenoma subtypes^48^. Data concerning its expression pattern in non-endocrine lesions of the sellar region are available for aCP^31^, but not for pCP and RCC. Therefore, the presented study is the first of its kind to demonstrate the distribution pattern and impact of EpCAM expression in a representative cohort of human non-endocrine sellar tumours.

Although all three entities are thought to be derived from the same cell of origin, and aCP and pCP are described as representing variants of the same tumour class, both EpCAM protein and gene expression levels revealed clear differences. Using immunohistochemistry, RCC and CP subtypes showed different staining patterns, pointing to a possible everyday application of EpCAM specific antibodies in the sometimes challenging differential diagnosis of cystic sellar lesions. Whereas all aCP and the majority of RCC cases showed EpCAM protein expression of variable intensity, the protein was never detectable within pCP. Most of the positive RCC showed only weak to moderate staining patterns, limited to the cell membrane of the cuboidal cells. EpCAM was already described to be expressed in aCP, but a detailed description of its distribution throughout the tumour was missing up to this point^31^. Based on our results, the protein is especially detectable in areas of stellate reticulum and surrounding regressive changes. In addition, our results confirm that EpCAM occurs in whirl-like cell structures, but that it is absent in clusters showing nuclear β-catenin accumulations. This is a very interesting observation because EpCAM was considered to be a target of Wnt/β-catenin^50^ and has further been described to interact with β-catenin in a Wnt-independent manner, affecting Wnt-target gene transcription^44^,^45^. However, the results we obtained using serial sections of aCP are in line with reports of budding cells in colorectal carcinomas, where the loss of membranous EpCAM was described in cells with nuclear translocation of β-catenin. This was also shown to significantly correlate with a higher extent of tumour budding, increased loco-regional spread, enhanced tumour recurrence and an increased migratory potential of tumour cells^51^.

The distinct EpCAM expression pattern surrounding β-catenin accumulating cell clusters was similar to that recently described for claudin-1^13^. This could hint at a potential interplay between EpCAM, claudins and other cell adhesion molecules in aCP as described earlier for T48 and Caco-2 cell lines^52^. EpCAM is further characterised as a versatile protein supporting numerous cell functions like homophile Ca^2+^ -independent cell adhesion^38^ and signal transmission with transcriptional regulation^32^,^44^, influencing other cell-cell adhesion proteins^39^,^42^ and playing a role in embryologic development^53^,^54^. EpCAM expression was already associated with migration, invasion and proliferation of neoplastic cells. Although the prognostic impact seems to be variable among different tumour types, an overall negative influence is clearly predominant^32^. In turn, overexpression often correlates with advanced tumour stages and poor overall survival rates^42^,^55^. In aCP, a strong EpCAM expression has been associated with a higher long-term risk of tumour regrowth^31^. We were not able to verify this conclusion in our patient cohort due to a lack of follow-up data.

Differential gene expression analysis among the CP subtypes confirmed our results obtained using immunohistochemistry. Overall, the group of aCP revealed significantly higher mRNA levels when compared to the pCP samples. The mechanisms regulating EpCAM gene expression in cancer are not fully understood. Recently, it was published that EpCAM can be influenced inter alia by EGF via ERK1/2 in epithelial ovarian cancer cell lines^56^. Activation of EGFR has been described in aCP^14^ and further studies will be required to demonstrate an interaction in aCP.

Surprisingly enough, significantly higher EpCAM staining levels were detectable in pediatric aCP compared to specimens obtained from adult patients. This is a very interesting observation because data concerning differences in the group of aCP dependent upon the age of onset had not been described so far. The fact that we were not able to verify the results on the mRNA level makes additional studies necessary to examine this phenomenon in detail.

In summary, EpCAM shows different expression patterns in CP subtypes and RCC, and may, therefore, be used as an additional diagnostic tool in the sometimes challenging differential diagnosis of sellar tumours. Highly significant differences in EpCAM gene expression levels between aCP and pCP confirm the protein data. An easy-to-handle algorithm to distinguish cystic sellar lesions in daily work using a three-marker panel (BRAF-VE1, EpCAM and β-catenin) is given in Table 2.

Our results point to a potential important role of EpCAM in the pathogenesis and biology of aCP, especially in early onset cases. Whether it may represent a new target for therapy has to be shown in additional studies examining its precise function and regulation.

## Methods

### Patient Cohort

For immunohistochemistry, we analysed surgical specimens from 74 patients with CP and 10 patients with RCC, all of which were obtained as formalin fixed tissue from the archive of the Department of Neuropathology at the University Hospital Erlangen-Nuremberg. The corresponding surgeries have taken place in the Departments of Neurosurgery at the University Hospital Erlangen-Nuremberg, the International Neuroscience Institute in Hannover, the University Hospital Hamburg-Eppendorf, the Evangelic Hospital Bielefeld-Bethel, the University General Hospital in Thessaloniki (Greece), the General Hospital in Vienna (Austria) and the Hospital “San Raffaele” in Milan (Italy). The neuropathology Department in Erlangen serves as a reference center for these samples. The group of CP was comprised of 10 tumour samples of the papillary and 64 of the adamantinomatous variant, representing the higher frequency of this CP subtype. Each tumour sample was classified according to World Health Organisation guidelines and only specimens containing sufficient amounts of the respective CP subtype were taken into account. Because reliable definitions concerning infiltrative growth are missing for CPs we used 2 independent classification systems to generate a clinical and histological cohort of invasive cases as previously described by Stache *et al*.^13^.

RCC samples were only chosen if additional molecular data excluding *BRAFV600E* or *CTNNB1* mutations was available confirming the diagnosis. Three RCC cases showed a distinct squamous metaplasia.

For gene expression analysis, we examined RNA extracted from 29 native CP samples showing sufficient amounts of vital tumour. The probes were derived from 19 patients with aCP and 10 patients with pCP. The clinical data is provided in Table 3 and detailed information concerning each tumour sample can be found in Supplementary Table 1 (S-Table 1).

### Ethical approval and informed consent

All experimental protocols using human samples were *approved by* the Ethical Committee of the University of Erlangen-Nürnberg. Informed consent from all subjects was obtained. All used methods were carried out *in accordance with* the approved guidelines of the Ethical Committee of the University of Erlangen-Nürnberg and in accordance with the Declaration of Helsinki. A declaration of consent for further scientific investigation is available from each patient for all specimens as prescribed by the local ethics committee of the Friedrich-Alexander-University Erlangen-Nuremberg.

### Immunohistochemistry

3 to 4 μm thick sections of formalin-fixed and paraffin-embedded surgical samples were prepared, then mounted on positively charged object slides (Superfrost, Menzel, Braunschweig, Germany), and dried at 37 °C overnight. Immunohistochemical staining was performed using a staining machine (Benchmark ULTRA IHC/ISH Staining Module; Ventana Roche; Illkirch, France) and the streptavidin-biotin-staining system Ventana DAB following the manufacturer’s recommendations. For the staining of EpCAM, we used a monoclonal anti-EpCAM antibody (MOC-31 clone specific against EpEX domain) generated in mice (1:200; CC1 standard; Cell Marque; Rocklin, CA, USA). β-catenin was detected by applying a monoclonal mouse-anti-β-catenin antibody (1:800; Clone 14; BD Biosciences; Franklin Lakes, NJ, USA). BRAF was examined by using a monoclonal antibody that selectively recognizes the BRAF V600E mutant epitope (BRAF V600E-specific clone VE1, Ventana, USA). The staining protocol included pretreatment with cell conditioner 1 (pH 8,4) for 64 min, incubation with VE1 at 36 °C for 16 minutes, primary antibody detection using the ultraView Universal DAB Detection Kit (Ventana), followed by counterstaining with hematoxylin for 4 minutes. Validation of this antibody has been previously reported in detail^57^. The staining procedures were verified by positive and negative controls (EpCAM: metastasis of an adenocarcinoma; β-catenin: colon carcinoma; negative control: omitting primary antibody).

### RNA Preparation

For the RNA extraction, shock frozen tissue samples were retrieved from our tissue bank (−80 °C). Total cellular RNA was extracted from 19 patients with aCP and 10 patients with pCP. Frozen sections of all tissues were microscopically reviewed to confirm tumour tissue content. RNA was isolated utilizing the RNeasy^®^ extraction kit (Qiagen). Subsequently, digestion with RNase-free DNase I (Invitrogen™, Carlsbad, CA, USA) and purification via RNeasy columns (Qiagen) were performed.

### Differential Gene Expression Analysis

Affymetrix U133 Plus2.0 expression array data were generated at the Microarray Department of the University of Amsterdam, the Netherlands, according to the manufacturer’s instructions. The MAS5.0 algorithm of the GCOS program (Affymetrix Inc) was used for normalization and assignment of detection p-values. Array quality was ensured by inspection of beta-actin and GAPDH 5′-3′ ratios as well as the percentage of present calls. Data was further investigated using the R2 microarray analysis suite (http://r2.amc.nl). The exact procedure is described more detailed elsewhere^58^. For this analysis, 19 adaCP and 10 papCP samples were included. Differential gene expression was performed using the log2 gene expression values of EPCAM.

### Scoring and Statistical Evaluation

EpCAM antigen expression was evaluated by two independent observers (VT and RB) using light microscopy (Olympus BX-50, Model U-MDOB, Olympus, Tokyo, Japan). In cases of discordant results, the specimens were re-evaluated on a double-headed microscope to attain consensus. Antigen expression was assessed as positive if specific staining at the surface membranes of tumour cells could be found. Only viable tumour areas were taken into account for quantity and intensity assessment, whereas areas with pronounced inflammatory and/or regressive tissue changes were omitted. The expression of EpCAM was evaluated by calculating a total immunostaining score (TIS) as described in similar studies^59^,^60^. A TIS is the product of a proportion score (PS), the estimated fraction of positive stained tumour cells (0 = 0%, 1 < 10%, 2 = 10–50%, 3 = 50–80%, 4 > 80%), and an intensity score (IS). The IS defines the estimated staining intensity (0 = no staining at all; 1 = weak staining; 2 = moderate staining; 3 = strong staining). The TIS ranges from 0 to 12 with only 9 possible values (0, 1, 2, 3, 4, 6, 8, 9, 12). The EpCAM total score was then divided into 3 scoring groups: scoring group 1 (S1, no expression, TIS = 0), scoring group 2 (S2, low/moderate expression, TIS = 1–4) and scoring group 3 (S3, strong expression, TIS > 4). Examples of each scoring group can be found in Fig. 1.

In order to analyse the association between different characteristics (e.g. age group or invasiveness) of the patient cohort and the EpCAM overexpression in aCP, the exact Fisher test was conducted to ensure reliable results amongst the relatively small number of samples. The results of the EpCAM gene expression were analysed for normal distribution using the Kolmogorov-Smirnov and the Shapiro-Wilks test. As Gaussian distribution could not be assumed for pCP, a Mann-Whitney test was performed for further statistical evaluation. All statistical analyses were conducted using IBM SPSS Statistics 23 (IBM, Armonk, NY, USA).

## Additional Information

**How to cite this article**: Thimsen, V. *et al*. EpCAM (CD326) is differentially expressed in craniopharyngioma subtypes and Rathke’s cleft cysts. *Sci. Rep.* **6**, 29731; doi: 10.1038/srep29731 (2016).

## Supplementary Material

Supplementary Information

## Figures and Tables

**Table 1:** EpCAM Protein Expression in aCP of Pediatric and Adult Patients.

	N	Children	Adults
**aCP total**	**64**	**23**	**41**
**No expression** (S1, TIS = 0)	0	0	0
**Low expression** (S2, TIS = 1–4)	39	9	30
**High expression** (S3, TIS > 4)	25	14	11

Children were defined as patients with ages ranging from 0–18 years. A significantly higher EpCAM expression could be observed in pediatric tumours when compared to findings in adult tumours [Fisher’s exact test (two-sided): *p* = *0.015*].

**Table 2:** Immunohistochemical profiles of cystic sellar lesions.

	BRAF (VE1)	EpCAM	nuclear β-catenin
pCP	−/+	−	−
aCP	−	+/++	−/+
RCC	−	−/+	−

**Table 3:** Summary of Clinical Data of patients with aCP, pCP and RCC included in immunohistochemical examination and gene expression analysis in this study.

	n	Female	Male	Children	Adults	Age mean (years)	Age range (years)
Immunohistochemistry							
aCP	64	25	39	23	41	31.3	1–72
pCP	10	3	7	0	10	45.8	19–62
RCC	10	8	2	0	10	47.6	22–68
Gene expression analysis							
aCP	19	5	14	8	11	32.9	6–66
pCP	10	3	7	0	10	42.9	36–58

Children were defined as patients with ages ranging from 0–18 years.
