# Supplementary material for: EpCAM (CD326) is differentially expressed in craniopharyngioma subtypes and Rathke’s cleft cysts
Source: Sci Rep. 2016 Jul 19;6:29731. doi: 10.1038/srep29731 (PMC4949472; doi:10.1038/srep29731)
Supplement: Supplementary Information [file srep29731-s1.pdf]

EpCAM (CD326) is differentially expressed in craniopharyngioma subtypes and Rathke's cleft cysts

Vivian Thimsen[1], Annett Hölsken[1], Michael Buchfelder[2], Jörg Flitsch[3], Rudolf Fahlbusch[4], Harald Stefanits[5], Marco Losa[6], David T.W. Jones[7], and Rolf Buslei[1*]

Supplementary material

Pat.	Sex	Age	Age Group	PS (0-4)	IS (0-3)	TIS	Scoring Group (S1-S3)	Log2-ratio	Invasive-ness	BRAF VE1	β-catenin nuclear
**pCP**											
pCP1	m	52	a	0	0	0	S1	-	no	neg	no
pCP2	m	19	a	0	0	0	S1	-	no	neg	no
pCP3	f	49	a	0	0	0	S1	6,1719	no	pos	no
pCP4	m	57	a	0	0	0	S1	-	-	-	no
pCP5	f	46	a	0	0	0	S1	6,7415	no	neg	no
pCP6	m	40	a	0	0	0	S1	-	yes	pos	no
pCP7	f	56	a	0	0	0	S1	-	-	neg	no
pCP8	m	31	a	0	0	0	S1	-	no	-	no
pCP9	m	62	a	0	0	0	S1	-	yes	-	-
pCP10	m	53	a	0	0	0	S1	-	-	-	-
pCP11	m	40	a	-	-	-	-	4,7970	-	neg	no
pCP12	m	36	a	-	-	-	-	7,6639	-	pos	no
pCP13	m	58	a	-	-	-	-	4,3923	-	pos	no
pCP14	m	42	a	-	-	-	-	4,9021	-	pos	no
pCP15	m	38	a	-	-	-	-	5,5849	-	neg	no
pCP16	m	45	a	-	-	-	-	4,0179	-	neg	no
pCP17	f	36	a	-	-	-	-	4,7655	-	neg	no
pCP18	m	39	a	-	-	-	-	4,6205	-	pos	no
**aCP**											
aCP1	m	48	a	1	2	2	S2	-	no	-	yes
aCP2	m	9	p	2	2	4	S2	-	-	-	yes
aCP3	m	50	a	2	2	4	S2	-	no	neg	yes
aCP4	f	3	p	3	3	9	S3	-	-	-	yes
aCP5	f	29	a	2	1	2	S2	-	yes	neg	yes
aCP6	f	33	a	2	3	6	S3	-	-	neg	yes
aCP7	f	14	p	3	3	9	S3	-	-	neg	yes
aCP8	m	8	p	1	1	1	S2	-	-	-	yes
aCP9	m	58	a	2	2	4	S2	-	-	-	yes
aCP10	f	2	p	2	1	2	S2	-	-	-	yes
aCP11	f	9	p	2	2	4	S2	-	no	neg	yes
aCP12	m	32	a	2	3	6	S3	-	no	-	yes
aCP13	m	28	a	1	2	2	S2	-	no	-	yes
aCP14	m	19	a	2	2	4	S2	-	no	-	yes
aCP15	m	22	a	2	3	6	S3	-	no	-	yes
aCP16	f	60	a	2	3	6	S3	-	-	-	yes
aCP17	m	56	a	1	2	2	S2	11,5439	no	neg	yes
aCP18	m	70	a	2	2	4	S2	-	no	neg	yes
aCP19	m	35	a	1	1	1	S2	-	no	-	yes
aCP20	f	35	a	1	1	1	S2	-	no	-	yes
aCP21	m	24	a	2	3	6	S3	-	no	-	yes
aCP22	m	15	p	1	2	2	S2	11,1487	no	neg	yes
aCP23	f	54	a	2	3	6	S3	-	yes	-	yes
aCP24	m	49	a	2	2	4	S2	-	-	-	yes
aCP25	m	13	p	3	3	9	S3	-	no	-	yes
aCP26	f	4	p	3	2	6	S3	-	yes	neg	yes
aCP27	f	47	a	1	1	1	S2	-	-	neg	yes

aCP28	m	35	a	1	2	2	S2	-	yes	-	yes
aCP29	f	4	p	2	2	4	S2	-	yes	-	yes
aCP30	f	11	p	2	3	6	S3	11,0883	yes	neg	yes
aCP31	m	39	a	2	3	6	S3	-	no	-	yes
aCP32	m	7	p	2	2	4	S2	12,2034	yes	neg	yes
aCP33	m	50	a	2	1	2	S2	11,2555	yes	neg	yes
aCP34	f	6	p	2	3	6	S3	-	-	neg	yes
aCP35	m	37	a	2	2	4	S2	-	yes	neg	yes
aCP36	f	58	a	1	2	2	S2	-	no	neg	yes
aCP37	f	63	a	2	3	6	S3	-	no	-	yes
aCP38	m	32	a	2	2	4	S2	11,4393	yes	neg	yes
aCP39	m	8	p	2	2	4	S2	-	yes	-	yes
aCP40	m	47	a	2	2	4	S2	-	yes	-	yes
aCP41	m	17	p	2	3	6	S3	-	yes	-	yes
aCP42	f	31	a	1	1	1	S2	-	-	-	yes
aCP43	f	47	a	2	2	4	S2	-	yes	neg	yes
aCP44	m	3	p	3	2	6	S3	-	no	neg	yes
aCP45	f	5	p	3	2	6	S3	-	no	neg	yes
aCP46	f	60	a	3	3	9	S3	-	-	-	yes
aCP47	m	72	a	3	3	9	S3	-	no	neg	yes
aCP48	f	16	p	3	2	6	S3	11,8929	no	neg	yes
aCP49	f	38	a	1	2	2	S2	-	yes	neg	yes
aCP50	m	39	a	3	3	9	S3	12,1675	yes	neg	yes
aCP51	m	1	p	3	3	9	S3	-	-	-	yes
aCP52	m	58	a	1	2	2	S2	11,0692	no	neg	yes
aCP53	m	48	a	1	2	2	S2	11,4343	no	neg	yes
aCP54	m	28	a	2	2	4	S2	-	yes	neg	yes
aCP55	f	7	p	3	2	6	S3	-	-	-	yes
aCP56	m	19	a	2	2	4	S2	-	-	-	yes
aCP57	m	9	p	2	2	4	S2	-	-	neg	yes
aCP58	f	54	a	1	3	3	S2	-	-	-	yes
aCP59	m	6	p	3	2	6	S3	-	-	-	yes
aCP60	m	67	a	1	1	1	S2	-	-	-	yes
aCP61	m	29	a	2	3	6	S3	-	-	-	yes
aCP62	f	58	a	2	2	4	S2	-	-	-	yes
aCP63	m	66	a	2	2	4	S2	-	yes	-	-
aCP64	m	7	p	2	2	4	S2	-	yes	-	-
aCP65	m	66	a	-	-	-	-	11,8101	-	neg	yes
aCP66	m	45	a	-	-	-	-	11,1118	-	neg	yes
aCP67	m	37	a	-	-	-	-	11,4005	-	neg	yes
aCP68	m	11	p	-	-	-	-	11,0488	-	neg	yes
aCP69	m	14	p	-	-	-	-	10,8565	-	neg	yes
aCP70	f	9	p	-	-	-	-	11,0990	-	neg	yes
aCP71	f	38	a	-	-	-	-	9,1119	-	neg	yes
aCP72	f	15	p	-	-	-	-	10,5132	-	neg	yes
aCP73	m	59	a	-	-	-	-	11,8353	-	neg	yes
**RCC**											
RCC1	m	46	a	2	1	2	S2	-	-	-	-
RCC2*	f	68	a	2	1	2	S2	-	-	-	-
RCC3	f	53	a	1	1	1	S2	-	-	-	-
RCC4	f	22	a	1	1	1	S2	-	-	-	-
RCC5*	m	56	a	1	1	1	S2	-	-	-	-
RCC6	f	53	a	2	1	2	S2	-	-	-	-
RCC7*	f	33	a	1	1	1	S2	-	-	-	-
RCC8	f	50	a	0	0	0	S1	-	-	-	-
RCC9	f	55	a	0	0	0	S1	-	-	-	-
RCC10	f	40	a	1	1	1	S2	-	-	-	-

S-Tab.1: Detailed clinical data, results of immunohistochemical EPCAM scoring, and log2-ratios of EPCAM gene expression of all patients included in this study. This overview includes gender, age, invasiveness, detailed immunohistochemical EpCAM scoring (PS= proportion score; IS= intensity score; TIS= total immunostaining score), log2-ratios from gene expression analysis, BRAF VE1 staining results, and presence of nuclear β-catenin accumulation within the tumour samples of all patients with aCP, pCP, and RCC included in

immunohistochemical evaluation and differential gene expression analysis of this study. (-) Not analysed. (*) RCC with squamous metaplasia.

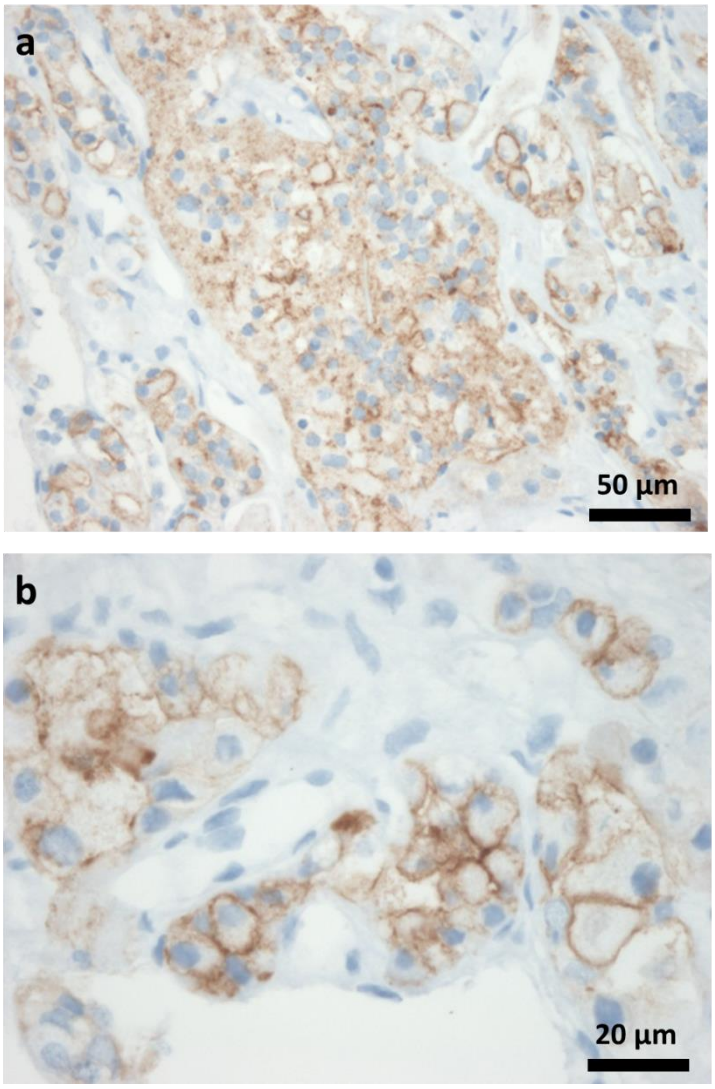

S-Fig. 1: Immunohistochemical EpCAM staining pattern of the anterior pituitary gland. Weak but homogeneous EpCAM immunostaining within the anterior pituitary gland, appearing predominantly at the cell surface membrane of endocrine cells.
